# High-Resolution Splenic Imaging: [^68^Ga]Ga-Oxine Red Blood Cell PET/CT for Differentiation of Splenosis Mimicking Malignant Lymphoma

**DOI:** 10.3390/tomography8060244

**Published:** 2022-12-12

**Authors:** Anke Werner, Martin Freesmeyer, Robert Drescher

**Affiliations:** Clinic of Nuclear Medicine, Jena University Hospital, 07747 Jena, Germany

**Keywords:** PET/CT, radiotracers, spleen, erythrocytes, oxine, oxyquinoline

## Abstract

The differentiation of splenic tissue from malignant lesions via imaging may be challenging, particularly considering aberrant or accessory lesions and diseases that are rarely encountered. Functioning splenic tissue can be identified using technetium-99m red blood cell (^99m^Tc-RBC) scintigraphy, but its sensitivity is limited and may not be available. We present the case of a patient in whom disseminated abdomino-pelvic splenosis was diagnosed using PET/CT with gallium-68-oxine-labeled RBCs. The method represents a feasible and probably superior alternative to splenic scintigraphy.

## 1. Introduction

Scintigraphic imaging of the spleen depends on the function of viable splenic tissue to remove aged, damaged, and/or otherwise altered erythrocytes from the blood stream. Spleen scintigraphy was introduced in the 1960s and gained importance with the introduction of technetium-99m (^99m^Tc) which allowed for examinations with low radiation exposure to patients and staff [1,2]. Red blood cells (RBCs) from venous blood are heat-damaged, labeled ex vivo, and reinjected intravenously. The accumulation of radioactivity in the body structure in question confirms the presence of functioning splenic tissue. Indications for splenic imaging include the identification of aberrant splenic tissue, the differentiation of splenic tissue from malignant lesions, and the evaluation of developmental anomalies of the spleen [3].

A trend in nuclear medicine imaging is the replacement of ^99m^Tc-based scintigraphy with positron emission tomography (PET)/computed tomography (CT) methods to utilize the higher spatial and temporal resolution of this modality. PET/CT with RBCs labeled with gallium-68-oxine ([^68^Ga]Ga-8-hydroxyquinoline) was first reported in 2021 and has since been in clinical use in our institution [4,5]. We report the case of a patient in whom disseminated abdomino-pelvic splenosis was diagnosed using spleen-specific PET/CT.

## 2. Case Presentation

A 67-year-old man underwent surgery for repair of an inguinal hernia. The clinical history of the patient included a surgical intervention after a traffic accident more than 40 years ago but was otherwise unremarkable. Due to complications, a laparotomy was necessary, and a postoperative CT was performed. A small bowel obstruction was ruled out, but the CT revealed multiple space-occupying lesions in the abdomen and pelvis with contrast enhancement. No visceral or bone lesions were identified. No spleen was present.

**Figure 1:**
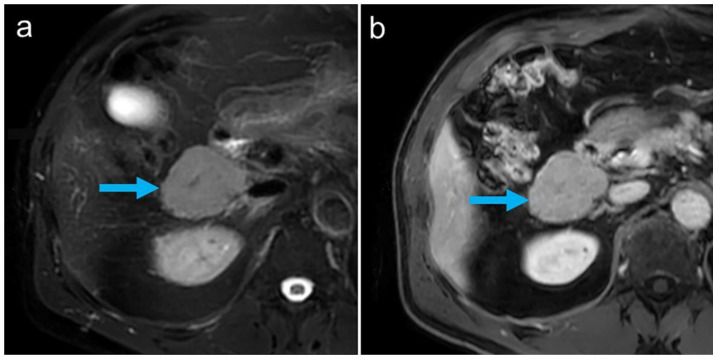
MR imaging showed well-defined masses (arrow: index lesion in the upper abdomen), moderately hyperintense on T2-weighted spectral presaturation with inversion recovery (SPIR) images (**a**) and with nearly homogeneous contrast enhancement on T1-weighted multiecho 2-point Dixon (mDIXON) sequences (**b**).

Initially, a malignant disease, particularly a lymphoma, was suspected, and magnetic resonance imaging (MRI) of the abdomen was performed for further evaluation. This confirmed the partially lobulated, well-defined lesions (Figure 1). In view of the previous surgery and the missing spleen, the differential diagnosis of multilocular ectopic splenic tissue (splenosis) was considered.

## 3. PET/CT Imaging

To avoid further interventions, a PET/CT with [^68^Ga]Ga-oxine-labeled RBCs was performed. Synthesis of [^68^Ga]Ga-oxine and RBC labeling procedures were adapted from [6,7]. A total of 8 mL of venous blood was taken from the patient in 2 mL of citrate anticoagulant buffer. The RBCs were separated from the plasma via centrifugation and heated to 48 °C for 10 min in order to induce heat denaturation and labeling with [^68^Ga]Ga-oxine. After three washing cycles with 0.9% saline, the labeled RBCs were resuspended. RBCs containing 188 MBq [^68^Ga]Ga-oxine were reinjected intravenously.

Imaging was performed on a Biograph mCT 40 scanner (Siemens Healthineers, Germany). After an unenhanced, low-dose CT for anatomical correlation and attenuation correction, an early dynamic PET was acquired continuously for 300 s, beginning with i.v. tracer injection. Static PET images (5 min per bed position) were acquired 10 and 25 min after tracer injection.

**Figure 2:**
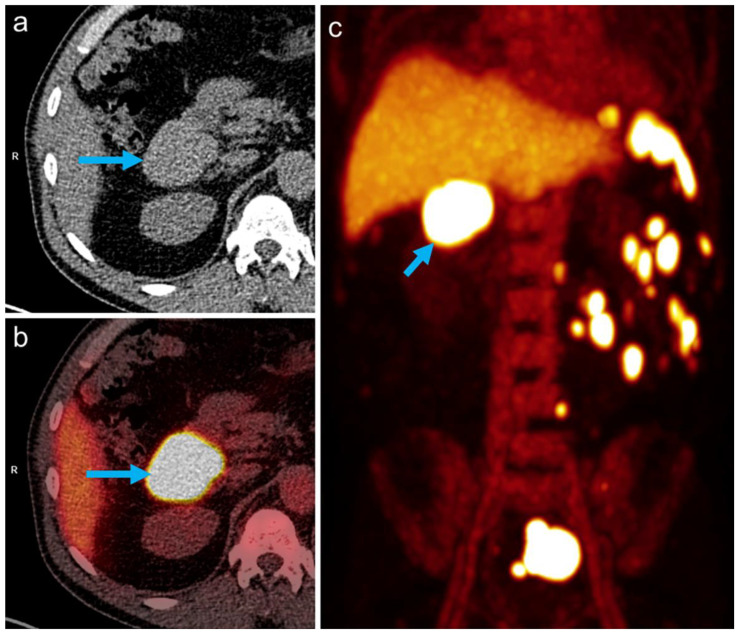
Multiple masses with high tracer uptake seen on RBC-PET/CT (**a**): non-contrast CT, (**b**): PET/CT fusion, (**c**): PET maximum intensity projection (MIP) image acquired 25 min after tracer injection).

All abdominal and pelvic masses showed a high tracer uptake increasing over time (Figure 2), with the highest uptake values measured in the subhepatic index lesion (Figure 1 and Figure 2, arrows): SUV_mean_: 69.1/94.1 at 10/25 min after tracer injection, respectively, showing an increase of 36% between the two time points. During the same interval, blood pool activity decreased (SUV_mean_: 9.6/6.8, −29%), indicating the removal of the labeled, heat-damaged RBCs from the blood by the functioning splenic tissue. The diagnosis of disseminated splenosis was confirmed. Since no symptoms were present and there was no evidence of an increased susceptibility to infections, no further treatment was necessary.

## 4. Discussion

This case illustrates the advantages of high-resolution [^68^Ga]Ga-RBC-PET/CT for the differentiation of splenic tissue from malignancies. The spatial resolution of PET/CT is approx. 2 mm, while that of ^99m^Tc single-photon emission computed tomography (SPECT) is 6–8 mm. PET/CT allows for voxel-based absolute uptake quantification and has the option of dynamic imaging for a time-resolved assessment of the tracer uptake.

In patients with small lesions, including accessory or maybe intrapancreatic spleens mimicking metastases of neuroendocrine tumors, this will be even more important and may avoid unnecessary surgical interventions with potentially severe complications [8,9,10]. It has been shown that the specificity of spleen scintigraphy is high, but its sensitivity for the detection of accessory spleens may be limited in non-splenectomized patients [11]. To date, for RBC-PET/CT, we have not detected a sink effect in patients with a native spleen, which may have limited the detectability of other small structures containing functioning splenic tissue.

The availability of PET/CT alternatives to evaluate various clinical issues is becoming increasingly important because the supply of molybdenum-99 (^99^Mo) and, thus, technetium-99m has been repeatedly disrupted in the past [12]. Currently, supply shortages of ^99^Mo are anticipated for Europe in November and December 2022, and these situations may become more frequent because the number of facilities producing ^99^Mo is decreasing [13].

RBC-PET/CT should be kept in mind as a problem solver in difficult cases of splenic disease, including splenosis and asplenia. It represents a feasible alternative to ^99m^Tc-based scintigraphic methods.
